# A Medical Causerie

**Published:** 1915-09-11

**Authors:** 


### A MEDICAL CAUSERIE.

Junior students of medicine are suffering just now from the confusion which results not infrequently from a multitude of counsellors. Many of them are within the limits of military age, and to these there has naturally come the supreme question which Lord Kitchener in his Guildhall speech addressed to the young men of the Empire. Does patriotism claim enlistment in the Army or the pursuit of medical studies? This is an issue which has been in debate in all the medical schools throughout the country. In the earlier months, when it was fondly believed that the war could not be a long experience, the question was not a very acute one, as six or twelve months' absence from the curriculum is not a great consideration, and the authorities were willing, so far as legally possible, to relax the rules in favour of the absentees. With the lengthening prospects of the war it soon became evident that the supply of doctors would be unequal to the demand, and the senior students were advised to complete their education and to take their diplomas; indeed, some of them were sent back from the Army for this purpose. This left the students of the earlier years in a somewhat agitated and uncertain state, and with a view to resolve the doubt a question was put to the Under-Secretary for War in the House of Commons in June last. The answer was that if large numbers of students in their first or second year joined the combatant ranks there would be after the war such a serious scarcity of doctors that to present such action as an obligation would not be in the interests of either the Army or the community. This appeared to settle the matter, at least for a time. But more recently, presumably as a result of the urgent appeals for recruits, doubts and uncertainties have again arisen, and under the influence of these Professor Halliburton, of King's College, addressed a letter direct to Lord Kitchener. The official reply is that the War Office "would be unwilling to suggest that junior students should be discouraged from taking combatant commissions." This indicates a complete change of view, and it must be assumed that the decision is a considered one. It means that the immediate necessities of the Army must take precedence of the more remote claims of the civil population. Yet the judgment has not escaped challenge, and it would seem to be advisable for some joint action to be taken by the medical schools. Until this course is adopted the unfortunate student is likely to be the victim of conflicting voices.

It is satisfactory to note that the War Emergency Committee has resolved to communicate with the governing bodies of the civil hospitals in reference to the advisability of abstaining from giving resident appointments to young men eligible for commissions in the R.A.M.C. The Hospital has repeatedly advised this policy. Yet advertisements for young men continue to appear in large numbers and without any indication that applications from candidates suitable for Army service will not be entertained. It is surely unseemly that junior members of the profession should be encouraged by the hospitals to turn a deaf ear to a claim which the highest authorities present as essential to the safety of the country. There is an opportunity for one or more of the hospitals openly to set a good example in this matter, by providing that no young man tarries there except for so long as is necessary to equip him for good work at the Front.

The question of admitting women students has been considered at Guy's Hospital, and the decision has been adverse to the proposal on the ground that there is not "at present" suitable accommodation for such a departure. This, it will be seen, does not express any opinion on the merits of the scheme, nor does it carry a note of finality. Accommodation which does not exist at present may possibly be provided in the future, and in view of what the future may bring the committee has probably been wise to leave an opportunity for revision. In the present state of opinion any medical school which instituted mixed classes would certainly run some measure of risk. Not a few men students object to such a scheme on the ground that in demonstrations, clinical visits, and so on, they would feel compelled to yield to women opportunities which they would not yield to their male colleagues. It is something to be in the front row, and when all have an equal chance those who get there can hold place without compunction. But most men would be reluctant to seize an advantage at the expense of a lady, even though the latter was intent on becoming a professional rival. The general impression seems to be that the objection to mixed classes would come from the women. But the other side of the house has some views on the matter, and the one just expressed is held in important quarters and cannot be denied as having some show of reason.

It is not often we hear from north of the Tweed a demand for legislative enactments which are already in operation in the southern portion of the island. But the claim for a Midwives Bill is supported by a most impressive company of gynæcological and obstetric experts, who point out that here also the influence of the war has created a situation which can only be described as a national emergency. The diminution in the ranks of the doctors means that a large amount of midwifery practice must fall into the hands of midwives and unqualified women, and many of these are absolutely untrained. Not less important is the consideration that, unless Parliament meets the situation, these women will remain entirely free from medical and official supervision such as has been organised in England under the Midwives Act. Mrs. Gamp, whatever her ancestry, was not, so far as we are told, a Scotswoman, but her professional descendants seem to have retreated to the North, and now professors and practitioners unite in asking that even there the eccentricities of the tribe shall be placed under supervision.

